# Determination of balance, fall risk, and kinesiophobia in individuals with Alzheimer’s Dementia

**DOI:** 10.3389/fpsyg.2025.1535440

**Published:** 2025-03-11

**Authors:** Oğuzhan Doğancı, Meral Sertel

**Affiliations:** ^1^Ministry of Internal Affairs the Rebublic of Turkey, Kastamonu District Governorship, Kastamonu, Türkiye; ^2^Health Sciences Faculty Physiotherapy and Rehabilitation Department, Bursa Uludag University, Bursa, Türkiye

**Keywords:** Alzheimer’s disease, balance, fall risk, kinesiophobia, gait

## Abstract

**Objective:**

This study aimed to determine balance, fall risk, and kinesiophobia in individuals with Alzheimer’s Dementia (AD).

**Methods:**

The study was completed with 18 AD and 18 healthy AD-free control group with early or moderate-stage AD diagnosed by a neurologist. Socio-demographic characteristics of the individuals were assessed using an evaluation form, and their balance was evaluated using the Tinetti Balance and Gait Assessment Test, Timed Up and Go Test, and Single Leg Standing Test. The Falls Risk Self-Assessment Scale (FRSAS) was used to assess the risk of falls. Kinesiophobia was assessed using the Tampa Scale for Kinesiophobia (TKS). Additionally, participants underwent the Mini-Mental State Examination (MMSE).

**Result:**

The mean age of individuals with AD was lower than that of healthy individuals, with means of 69 ± 3.66 years and 65.4 ± 4.10 years, respectively (*p* = 0.012). The Tinetti balance (*p* = 0.005), Tinetti gait (*p* < 0.001), Tinetti total (*p* < 0.001), and the Mini-Mental State Examination (MMSE) (*p* < 0,001) scores were lower in AD individuals relative to controls. The FRSAS (*p* < 0.001) scores were higher in AD individuals relative to controls. The TKS scores were found to be similar between individuals with AD and the control group (*p* = 0.860).

**Conclusion:**

It was found that individuals with Alzheimer’s disease (AD) have poorer balance and a higher risk of falls compared to healthy individuals. In light of these results, balance assessments should be included when developing rehabilitation protocols for individuals with AD. Treatment protocols designed for this patient group must incorporate balance-specific exercise and training programs. Additionally, individual and environmental preventive measures should be implemented to reduce the risk of falls in individuals with AD.

**Clinical trial registration:**

Clinical Trial Number: NCT05201768.

## Highlights

- Alzheimer’s disease have poorer balance
- Alzheimer’s disease have a higher risk of falls
- Individual and environmental preventive measures should be implemented to reduce the risk of falls in individuals with Alzheimer’s disease.

## 1 Introduction

As a result of the gradual increase in life expectancy in the population, the proportion of the elderly has increased. The reasons for the increase in this rate are the development of modern treatment methods, the increase in the age of death due to the increase in socioeconomic and cultural levels, and the decrease in fertility (Gürer et al., 2019). Along with this trend, there has also been an increase in age-related diseases. One of these diseases is Dementia, of which Alzheimer’s is the most common form. After the age of 65, the incidence of AD increases, and it is more prevalent in women than in men (Işık and Tanrıdağ, 2009).

AD is a chronic neurodegenerative disease for which there is no proven cure (Jia et al., 2020). First described in 1907, Alzheimer’s disease has multiple etiologies, but its exact causes remain unclear. The etiological factors contributing to Alzheimer’s disease are complex and multifactorial, involving a combination of genetic, environmental, and lifestyle influences (Passeri et al., 2022). This neurodegenerative disorder is estimated to affect 50 million people worldwide, and when considering their families, it indirectly impacts the lives of tens of millions of individuals (Alzheimer's Association, 2019; Rostagno, 2022).

In individuals with Alzheimer’s, the aging process, along with degeneration of the vestibular and visual systems, leads to a loss of muscle strength, proprioception, and joint mobility (Black and Wood, 2005). Falls are a significant health concern in individuals with AD (Buchner and Larson, 1987; Dyer et al., 2020). Falls are associated with serious injuries and loss of confidence in all older adults, but particularly in those with dementia, and are linked to increased morbidity and mortality (Allan et al., 2009). Individuals with AD fall 2 to 3 times more frequently than their cognitively healthy peers of the same age (Ries et al., 2015).

Kinesiophobia is defined as “an excessive and irrational fear of physical movement and activity due to a sense of vulnerability following painful injury or re-injury” (Devecchi et al., 2022; Kori, 1990). Individuals with kinesiophobia believe that avoiding movement is an appropriate response, which leads to harmful behaviors and a decline in overall functional ability (Lethem et al., 1983; Vlaeyen et al., 2016). Kinesiophobia in individuals can develop either directly from experiences such as pain or through social observation (Güzel et al., 2021). It has been observed that those who have fallen at least once in the past year tend to avoid physical activity more than those who have not fallen (Bertera and Bertera, 2008). Since patients with kinesiophobia believe that movement will cause re-injury or increase pain, they often reduce their physical activity and avoid it for prolonged periods (Goubran et al., 2024). This extended avoidance can lead to physical deconditioning, impaired cardiovascular health, and reduced mobility, all of which are critical factors in maintaining brain health and cognitive function. In the context of Alzheimer’s disease, this avoidance behavior may exacerbate cognitive decline by limiting the neuroprotective benefits of physical activity, such as improved blood flow, enhanced neurogenesis, and reduced neuroinflammation. Addressing kinesiophobia in at-risk populations or individuals with AD could play a vital role in breaking the cycle of physical inactivity and cognitive deterioration (Vranceanu et al., 2023).

Kinesiophobia is more common in older adults due to factors specific to aging, such as fear of falling, physical fragility, and chronic pain. Fear of falling causes older adults to lose their confidence in physical activities. As a result, it can trigger a sedentary lifestyle in the elderly. At the same time, chronic diseases and loss of muscle strength that increase with age facilitate the development of kinesiophobia. This may lead to further reduction of mobility in older individuals and difficulty in independent living (Scheffer et al., 2008). Kinesiophobia is an important factor that directly affects the mobility and physical performance of older adults. This condition can cause problems such as slower walking speed, decreased balance and limited range of motion in the elderly (Crombez et al., 1999). In addition to cognitive impairment, motor disorders and postural instability are also manifested in Alzheimer’s disease (Fried et al., 2001). The relationship between kinesiophobia and AD is an emerging area of interest, particularly in the context of how physical activity impacts cognitive health. While there is limited direct research on kinesiophobia in individuals with AD, several connections can be drawn.

However, kinesiophobia may prevent individuals, particularly older adults, from engaging in physical activity. This avoidance behavior can accelerate physical and cognitive decline, indirectly exacerbating AD progression or risk. For example, reduced movement leads to vascular dysfunction, slower metabolic clearance of amyloid-beta, and decreased neuroplasticity, all of which accelerate neurodegeneration in AD (Liu et al., 2024). Upon examining the relevant literature, it was found that previous studies only assessed balance in individuals with AD. Therefore, additional studies are needed to assess balance, fall risk, and kinesiophobia together. Studies on balance and fall risk in AD often focus on physical or motor impairments without integrating psychological aspects like kinesiophobia. Research on kinesiophobia has primarily centered on musculoskeletal conditions (e.g., chronic pain, osteoarthritis) or post-injury populations rather than neurodegenerative disorders. For that reason, this study aimed to evaluate balance, fall risk, and kinesiophobia in individuals with AD and compare them with healthy individuals.

## 2 Methods

### 2.1 Participants

**Figure 1:**
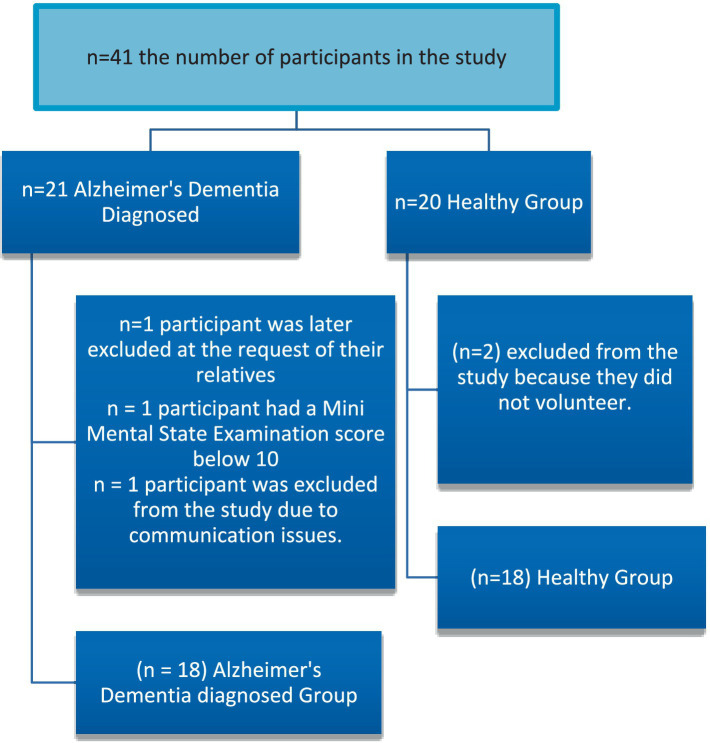
Study flowchart.

This study was conducted between January 2022 and April 2023, involving 21 individuals diagnosed with early or moderate-stage AD by a neurologist at Halil Şıvgın Çubuk State Hospital, and 20 control group. According to the MMSE, 15 individuals had moderate AD, and 3 individuals had mild AD. One individual diagnosed with AD was excluded from the study at the request of their relatives, while another was excluded due to communication issues and a diagnosis of advanced-stage AD. Among the control group, 2 individuals were not included as they declined to participate (Figure 1). Individuals in the control group were recruited for this cross sectional study through snowball sampling (Özsoy, 2014).

The study was approved by the Non-Interventional Clinical Research Ethics Committee of the Faculty of Health Sciences at Kırıkkale University on October 21, 2021 (Decision No: 2021.10.03). Permission was also obtained from the Chief Physician’s Office of Halil Şıvgın Çubuk State Hospital on August 10, 2021. Furthermore, the study was conducted in accordance with the principles of the Helsinki Declaration. This study was derived from a master’s thesis.

In the study, 18 individuals with AD in early or middle stage were diagnosed by a neurologist, and 18 controls were evaluated. G*Power (version 3.1.9.7, Universitat Düsseldorf, Düsseldorf, Germany) was used for post-hoc power analysis and the effect size was calculated from the total score of the Tinetti Balance and Gait Test in Alzheimer’s patients and healthy elderly individuals. According to the analysis, when the statistical significance of the two-way hypothesis test alpha was taken as 5%, and the confidence interval was 95%, the effect size was found to be 1.40, and the power of the study (1-β) was found to be 99%. The individuals participated in the study voluntarily.

The inclusion criteria for individuals with Alzheimer’s disease in the study were as follows: aged between 50 and 80 years, ability to walk independently, a diagnosis of early or middle-stage Alzheimer’s disease made by a physician, and willingness to participate in the study. For individuals in the control group, the criteria included: aged between 50–80 years, ability to walk independently, willingness to participate in the study, and no diagnosis of Alzheimer’s disease. Both the AD and control Individuals who had any barriers to physical activity, those who had undergone surgery in the past 6 months, those who refused to participate in the study, individuals diagnosed with late-stage Alzheimer’s disease, and those using medications that negatively affect balance and walking, having psychiatric diseases (depression, panic attacks etc.) or having neurological diseases or having orthopedic diseases were excluded from the study.

The individuals included in the study were evaluated with the assessment form prepared by the investigator. During the assessment, the individuals were under the supervision of a physiotherapist. In the assessment form, gender, age, height, weight, body mass index, smoking, alcohol use, marital status, educational status, medications and chronic diseases and type were recorded to determine the socio-demographic characteristics of the individuals.

The study was completed with 18 individuals with AD and 18 controls. The physical characteristics of the study participants are shown in Table 1.

**Table 1:** Physical characteristics of the participants.

Physical Characteristics	Control group (*n* = 18)		Alzheimer dementia group (*n* = 18)			
	Mean ± SD	Median (%25–75 IQR)	Mean ± SD	Median (%25–75 IQR)	U^#^	P^*^
Age (years)	65.4 ± 4.10	65.50(63–67.25)	69 ± 3.66	69(66–72.25)	83.50	0.012^#^
Height (cm)	167.88 ± 7.69	170(160–173.50)	163.66 ± 9.89	168(153–172)	119.50	0.181^#^
Body Weight (kg)	79.61 ± 7.10	80(75–83.25)	77.88 ± 6.12	79(79–84)	143.50	0.563^#^
BMI (kg/m^2^)	28.39 ± 3.49	27.68(25.83-31.65)	29.25 ± 3.19	28.36(27.25-31.43)	135.50	0.436^#^

*p < 0.05; ^#^U: Mann–Whitney U test, *n*: Number of Participants; IQR, Interquartile Range, SD, Standard Deviation, BMI, Body Mass Index.

The mean age of individuals with AD was higher than that of healthy individuals, with means of 69 ± 3.66 years and 65.4 ± 4.10 years, respectively (*p* = 0.012). Height (*p* = 0.181), body weight (*p* = 0.563), and BMI (*p* = 0.436) values were similar between the groups (Table 1).

There was no difference between AD and Control groups in terms of gender, marital status, education level, chronic disease status, drug, smoking, and alcohol use (Table 2).

**Table 2:** Socio-demographic characteristics of the participants.

Variable		Alzheimer Dementia Group (*n* = 18)		Control Group (*n* = 18)		Total (*n* = 36)		*X*^2^(df)	p*
		Number of Participants	%	Number of Participants	%	Number of Participants	%		
Gender	Male	11	61.1	10	55.6	21	58.3	0.114(1)	0.735
	Female	7	38.9	8	44.4	15	41.7		
Marital Status	Married	11	61.1	14	77.8	25	69.4	1.178(1)	0.278
	Single	7	38.9	4	22.2	11	30.6		
Education Level	Primary School and Below	13	72.2	9	50.0	22	61.1	8.056(6)	0.234
	High School and Above	5	27.8	9	50.0	14	38.9		
Chronic Disease Status	None	2	11.1	4	22.2	6	16.7	11.00(11)	0.443
	Present	16	88.9	14	77.8	30	83.3		
Chronic Disease Type	Diabetes (DM)	2	11.2	2	11.2			11.00(11)	0.443
	Hypertension (HT)	5	27.8	5	27.8				
	DM + HT	6	33.4	5	27.8	36	100		
	Cardiovascular Disease	3	16.8	2	11.2				
	None	2	11.2	4	22.2				
Medication Use Status	None	1	5.6	4	22.2	5	13.9	11.133(11)	0.432
	Present	17	94.4	14	77.8	31	86.1		
Type of Drug (Daily)	Anti-DM	2	11.2	2	11.2			11.133(11)	0.432
	Anti-HM	5	27.8	5	27.8				
	Anti-DM + Anti-HT	6	33.4	5	27.8	36	100		
	CVD	3	16.8	2	11.2				
	None	1	5.6	4	22.2				
Smoking Status	Smoking	8	44.4	8	44.4	16	44.4	0.000(1)	1.000
	Non-Smoking	10	55.6	10	55.6	20	55.6	1.029(1)	0.310
Alcohol Use Status	Alcohol Use	1	5.6	-	-	1	2.8		
	No Alcohol Use	17	94.4	18	100.0	35	97.2		

*X*^2^:Chi-square test; df, degrees of freedom; *p < 0.05 indicates significance.

It was found that the mean values of the Mini Mental Test measurements were lower in the AD group than in the Control Group [*F*(1,34) = 16.53; *p* = 0.001] (Table 3).

**Table 3:** Comparison of cognitive status, fall risk, and kinesiophobia in Alzheimer dementia and control group.

Variable	Alzheimer dementia group (*n* = 18)		Control group (*n* = 18)				
	Mean ± SD	Rank mean	Mean ± SD	Rank mean	*p*(df)	p*	*η*_p_^2^
MMSE	17.33 ± 3.14	12.14	24.00 ± 3.46	23.44	16.53(1.34)	<0.001*	0.501
TKS Score	46.50 ± 3.96	18.86	46.22 ± 5.43	18.14	0.15(1.34)	0.860	0.009
FRSAS	7.44 ± 1.98	23.81	3.89 ± 3.27	13.19	9.74(1.34)	<0.001*	0.371

*p < 0.05; ** Analysis of Covariance, with age variable controlled. (ANCOVA); *n*: Number of Participants; η_p_^2^: partial eta squared; df, degrees of freedom; SD, Standard deviation; MMSE, Mini Mental State Examination; TKS, Tampa Kinesiophobia Scale; FRSAS, Fall Risk Self-Assessment Scale.

### 2.2 Tests administered

Tinetti Balance and Gait Assessment Test, Timed Up and Go Test and Single Leg Standing Test were used to assess balance. The TKS was used to assess kinesiophobia, and the Fall Risk Self-Assessment Scale was used to assess fall risk. Mini Mental State Examination was also performed.

Tinetti Balance and Gait Assessment test, evaluates balance ability and walking under two main categories. The first 9 questions are related to balance, and the total score from these questions is the balance score. The next 7 questions are related to walking, and the total score from these questions is the walking score (Savaş and Akçiçek, 2010). The required equipment includes an armless chair, a stopwatch, and a 15-meter walking area. The test is completed in 10–15 min. Each item is scored with three possible values: 0, 1, or 2. A score of 2 indicates that the movement is performed correctly, 1 indicates that the movement is performed with adaptations, and 0 indicates that the movement cannot be performed. The total score is the sum of the balance score and walking score. The total balance score is 16; scoring below 11 points indicates a risk of falling. The total walking score is 12; scoring below 8 points indicates a risk of falling (Tinetti, 1986). The validity and reliability of the Turkish version of the Tinetti Balance and Gait Assessment, developed to measure balance and gait in patients with chronic renal failure, was conducted. The internal consistency (Cronbach alpha) was 0.90. ICC score for the test–retest reliability coefficient was 0.97. The findings indicate that the Turkish version is a reliable and valid tool for assessing balance and gait problems in end-stage disease patients who underwent hemodialysis (Ağırcan, 2009).

Timed Up and Go Test was developed by Podsiadlo and Richardson (1991) (Arnold and Faulkner, 2007; Thrane et al., 2007). The purpose of this test is to assess functional mobility and dynamic balance (Kaya et al., 2012; Tuncay et al., 2011). A chair and a stopwatch are sufficient to perform this test. During the test, the individual is given the following commands in sequence: “Start, stand up from the chair, walk at a normal pace to the line ahead, turn back, walk at a normal pace towards the chair, and sit down.” The total time is recorded in seconds. If the elderly individual takes more than 12 s to complete the test, it indicates a risk of falling (Blankevoort et al., 2013; Podsiadlo and Richardson, 1991).

Single Leg Standing Test is a test used by physical therapists to assess stability (Hawk et al., 2006). The single leg stance test is commonly used to identify functional decline and has been shown to be sensitive in clinical applications (Hawk et al., 2006). During the test, the participant is asked to lift one leg while keeping their eyes open, without letting the lifted leg touch the support leg. The test is ended when the lifted leg touches the support leg, when the foot makes contact with the ground, when there is any hopping or bouncing, or when the participant touches anything nearby for support (Stevenson and Garland, 1996). The time the individual remains on one leg is recorded in seconds.

The FRSAS is an assessment tool used to evaluate the risk of falls in older adults. It consists of 13 items that assess fall risk in elderly individuals. Responses are given as “yes” (1 point) or “no” (0 points), and individuals scoring 4 points or more are classified as having a high risk of falling. Sertel et al. conducted the Turkish validity and reliability study of the FRSAS in older adults. The ICC value of the FRSAS was found to be 0.999 (95% CI; 0.995–1.000 excellent agreement). Cronbach’s alpha coefficient was computed as 0.872.The scale’s internal consistency was observed to be very high. Sertel et al. demonstrated that the Turkish version of the FRSAS was valid and reliable in older adults (Sertel et al., 2020).

The TKS was originally developed by Miller et al. (1991), although it was not published. Vlaeyen and colleagues republished the original 17-item scale in 1995 with the permission of the original developers. The TKS is a 17-item scale designed to measure fear of movement/reinjury. The scale includes parameters related to work-related activities, injury/reinjury, and fear-avoidance (Vlaeyen et al., 1995). Yilmaz et al. conducted the Turkish validity and reliability study of TKS in low back-neck patients. The test–retest reliability was found to be 0.806 (95% CI = 0.720–0.867). Test–retest reliability of the Turkish version of the TKS was found to be excellent, and it was seen that the tool could be used in clinical settings (Yilmaz et al., 2011).

The MMSE was validated for diagnosing mild dementia in the Turkish population in 2002 (Güngen et al., 2002). The MMSE consists of 11 questions and is scored out of a total of 30 points. The questions in the test fall under five main categories: orientation, registration memory, attention and calculation, recall, and language. The total score is calculated by summing the scores obtained from each category. A score of 26–30 is considered within normal limits, a score between 24 and 26 is questionable; a score between 21 and 24 indicates mild cognitive impairment; a score between 10 and 20 corresponds to a moderate cognitive impairment and finally, a score between 0 and 9 suggests a severe cognitive impairment (Çuhadar et al., 2006). There is no time limit for the individual being assessed when taking the test.

### 2.3 Procedure

The assessments were performed face-to-face by an experienced physiotherapist in the clinic. The researchers prepared an assessment form that included the scales. The tests were applied to all older adults in the same order. While applying the tests, each individual’s fatigue and suitability for the test were considered. Since the tests were administered individually, the best time to test each older adult was determined when they were not tired, felt vigorous and well. The tests were administered face-to-face by OD in paper and pencil format for approximately 20 min for each individual.

### 2.4 Statistical analyses

Descriptive statistics for categorical variables (demographic characteristics) were presented as frequencies and percentages. The normal distribution of continuous variables was checked using the “Shapiro–Wilk Test.” The homogeneity of variances was tested using Levene’s test. Descriptive statistics for continuous variables were given as mean ± standard deviation for data showing a normal distribution and as median (min–max) values for data not showing a normal distribution. For the comparison of two independent groups without a normal distribution, the “Mann–Whitney U Test” was performed. Analysis of Covariance was used to examine the differences between the two groups by controlling age. “The Pearson Chi-Square test” was used to compare categorical variables between groups such as gender, marital status, education level, chronic disease status, type of chronic disease, drug use, drug type, cigarette, and alcohol use. Spearman correlation coefficients were used to obtain the correlation coefficients. In R correlation coefficients, if the r coefficient was 0.00–0.29, it was accepted as a very weak relationship; if it was 0.30–0.64, it was accepted as a moderate relationship; and if it was 0.65–0.84, it was accepted as a strong relationship. In all calculations and interpretations in the study, a significance level of *p* < 0.05 was considered, and hypotheses were formulated as two-sided. The statistical analysis of the data was performed using the SPSS v26 (IBM Inc., Chicago, IL, USA) statistical software package.

## 3 Results

### 3.1 Tinetti balance and gait tests

Table 4 presents the comparison of measurement results for the Tinetti balance, Tinetti gait, and Tinetti total tests between individuals with Alzheimer’s disease (AD) and control group. It was found that the rank means and average values for the Tinetti balance [*F*(1,34) = 6.37; *p* = 0.005], Tinetti gait [*F*(1,34) = 19.91; *p* = 0.001] and Tinetti total [*F*(1,34) = 10.27; *p* = 0.001] measurements were lower in the AD group compared to the Control Group. When the results of the study ANCOVA analysis were examined, the Partial Eta Squared (Tinetti-Balance = 0.141; Tinetti-Gait = 0.298; Tinetti-Total = 0.198) showed that a large effect was observed (Table 4).

**Table 4:** Comparison of Tinetti Balance, Tinetti Gait, and Tinetti Total test results between Alzheimer dementia and control groups.

Variable	Alzheimer dementia group (*n* = 18)		Control group (*n* = 18)				
	Mean ± SD	Rank Mean	Mean ± SD	Rank Mean	*F*(df)	p*	η_p_^2^
Tinetti Balance Score	15.28 ± 4.28	13.64	19.61 ± 3.79	23.56	6.37(1.34)	**0.005**	0.279
Tinetti Gait Score	5.17 ± 1.79	11.58	7.89 ± 1.45	25.42	19.91(1.34)	<0.001*	0.547
Tinetti Balance and Gait Total Score	20.44 ± 5.70	12.72	27.50 ± 5.06	24.28	10.27(1.34)	<0.001*	0.384

*p < 0.05; ** Analysis of Covariance, with age variable controlled (ANCOVA); *n*: Number of Participants; SD: Standard deviation; η_p_^2^: partial eta squared; df: degrees of freedom.

### 3.2 Timed up and go test

It was found that the rank means and average values for the Timed Up and Go Test [*F*(1,34) = 8.82; *p* = 0.001] measurements were higher in the AD group compared to the Control Group (Table 5).

**Table 5:** Comparison of timed up and go test and single leg stand test results between alzheimer dementia and control groups.

Variable	Alzheimer dementia group (*n* = 18)		Control group (*n* = 18)				
	Mean ± SD	Rank mean	Mean ± SD	Rank mean	*F*(df)	p*	η_p_^2^
Timed Up and Go Test (seconds)	29.17 ± 15.22	24.64	13.79 ± 4.93	12.36	8.82(1.34)	0.001*	0.348
Single Leg Stand Test (seconds)	6.00 ± 5.70	12.67	14.33 ± 7.72	24.33	9.44(1.34)	0.001*	0.364

*p < 0.05; Analysis of Covariance, with age variable controlled (ANCOVA); *n*: Number of Participants; SD: Standard deviation; η_p_^2^: partial eta squared; df: degrees of freedom.

### 3.3 Single leg stand test

It was found that the rank means and average values for the Single Leg Stance Test measurements were lower in the AD group compared to the Control Group [*F*(1,34) = 9.44; *p* = 0.001]. When the results of the study ANCOVA analysis were examined, the Partial Eta Squared (TUG = 0.243; Single Leg Stance Test = 0.171) showed that a large effect was observed (Table 5).

### 3.4 Fall risk

The rank mean values of FRSAS [*F*(1,34) = 9.74; *p* = 0.001] measurements were found to be higher in the AD group than in the Control Group (*p* < 0.05) (Table 3). When the results of the study ANCOVA analysis were examined, the Partial Eta Squared (FRSAS = 0.206) showed that a large effect was observed (Table 3).

### 3.5 Kinesophobia

It was found that the mean values of the TKS [*F*(1,34) = 0.15; *p* = 0.860] measurements were found to be higher in the AD group than in the Control Group (*p* < 0.05) (Table 3).

### 3.6 Correlations between Tinetti balance-gait-total, time up and go test, single leg stand test, fall risk and Kinesiophobia in Alzheimer patients and controls

The relationship between the measurement results of the individuals in the Alzheimer’s Dementia group is shown in Table 6. Accordingly, there was a negative relationship between MMSE and age, which suggests that older participants had lower MMSE scores. A positive moderate relationship between MMSE and Tinetti Gait and Tinetti Total suggests that good balance was associated with higher MMSE scores. A negative moderate relationship between MMSE and FRSAS indicates that those with a high risk of falling had lower MMSE scores.

**Table 6:** Correlation between test results of individuals in the Alzheimer’s Dementia group.

		(1)	(2)	(3)	(4)	(5)	(6)	(7)	(8)
Age (1)	*r*	1.000							
	*p*								
MMSE (2)	*r*	**−0.671** ^**^	1.000						
	*p*	**0.002**							
Tinetti Balance (3)	*r*	−0.095	0.434	1.000					
	*p*	0.707	0.072						
Tinetti Gait (4)	*r*	−0.359	**0.537** ^*^	**0.678** ^**^	1.000				
	*p*	0.0143	**0.022**	**0.002**					
Tinetti Total (5)	*r*	−0.280	**0.514** ^*^	**0.942** ^**^	**0.820** ^**^	1.000			
	*p*	0.261	**0.029**	**0.000**	**0.000**				
Timed Up and Go Test (6)	*r*	−0.814	−0.434	**−0.796** ^**^	**−0.692** ^**^	**−0.844** ^**^	1.000		
	*p*	0.060	0.072	**0.000**	**0.001**	**0.000**			
Single Leg Standing Test (7)	*r*	−0.249	0.468	**0.831** ^**^	**0.818** ^**^	**0.902** ^**^	**−0.897** ^**^	1.000	
	*p*	0.320	0.050	**0.000**	**0.000**	**0.000**	**0.000**		
TKS (8)	*r*	0.006	−0.432	−0.296	−0.415	−0.356	**0.501** ^*^	−0.376	1.000
	*p*	0.982	0.074	0.233	0.087	0.147	**0.034**	0.124	
FRSAS	*r*	0.093	**−0.479** ^*^	**−0.586** ^*^	**−0.756** ^**^	**−0.675** ^**^	**0.596** ^**^	**−0.697** ^**^	0.353
	*p*	0.715	**0.044**	**0.011**	**0.000**	**0.002**	**0.009**	**0.001**	0.150

*p < 0.05 **p < 0.001; *r* = Spearman Korelasyon Katsayısı; MMSE, Mini Mental State Examinitation; TKS, Tampa Kinesiophobia Scale Score; FRSAS, Fall Risk Self-Assessment Scale.

Significant strong relationships were identified between the Tinetti Balance, Tinetti Gait and Tinetti Total scores, and the Timed Up and Go Test, as well as between the Single Leg Standing Test and Tinetti Balance, Tinetti Gait and Tinetti Total scores, and the Timed Up and Go Test. Accordingly, there was a strong relationship between static and dynamic balance tests, which showed that if elderly individuals’ dynamic balance scores were high, their static balance scores were also high (Table 6).

In the control group, high positive correlations were found between MMSE and Tinetti Balance, Tinetti Gait, Tinetti Total, and Single Leg Standing Tests, suggesting that good balance was associated with higher MMSE scores. There were high negative correlations between MMSE and the Timed Up and Go Test, FRSAS, and TKS, suggesting that a high risk of falling and kinesiophobia were associated with lower MMSE scores. In addition, a high negative correlation was found between the Timed Up and Go test and the FRSAS, as well as the TKS, suggesting that participants who had higher scores on the Timed Up and Go test had lower scores on the FRSAS and TKS.

A positive high correlation was found between TKS and Timed Up and Go Test, suggesting that a high kinesiophobia was related worse functional mobility and dynamic balance scores. Negative moderate correlations were found between Tinetti Balance, Tinetti Gait, Tinetti Total, FRSAS, and Single Leg Standing Test, suggesting that a high risk of falling and kinesiophobia was related to worse balance scores. A negative moderate correlation was found between FRSAS and Single Leg Standing Test, suggesting that a high risk of falling was related worse static balance scores. A positive high correlation was found between FRSAS and Tinetti Balance, Tinetti Gait, Tinetti Total scores; and a high positive correlation was found between Timed Up and Go Test, which suggests that a high risk of falling was associated with worse balance scores (Table 7).

**Table 7:** Correlation between test results of individuals in the control group.

		(1)	(2)	(3)	(4)	(5)	(6)	(7)	(8)
Yaş (1)	*r*	1.000							
	*p*								
MMSE (2)	*r*	−0.194	1.000						
	*p*	0.441							
Tinetti Balance (3)	*r*	−0.318	0.836**	1.000					
	*p*	0.199	**<0.001**						
Tinetti Gait (4)	*r*	−0.305	**0.866** ^**^	**0.910** ^**^	1.000				
	*p*	0.219	**<0.001**	**<0.001**					
Tinetti Total (5)	*r*	−0.327	0.839**	0.997**	0.928**	1.000			
	*p*	0.186	**<0.001**	**<0.001**	**<0.001**				
Timed Up and Go Test (6)	*r*	0.187	−0.637*	−0.740**	−0.635**	−0.743**	1.000		
	*p*	0.458	**0.004**	**<0.001**	**0.005**	**<0.001**			
Single Leg Standing Test (7)	*r*	−0.369	0.636**	0.730**	0.575*	0.722**	-0.649**	1.000	
	*p*	0.132	**0.005**	**<0.001**	**0.013**	**<0.001**	**0.004**		
TKS (8)	*r*	0.033	-0.588*	-0.677**	-0.579*	-0.673**	0.804**	-0.549*	1.000
	*p*	0.897	**0.010**	**0.002**	**0.012**	**0.002**	**<0.001**	**0.018**	
FRSAS	*r*	0.175	-0.692**	-0.713**	-0.711**	-0.723**	0.806**	-0.550*	0.586*
	*p*	0.486	**0.001**	**<0.001**	**<0.001**	**<0.001**	**<0.001**	**0.018**	**0.011**

*p < 0.05 **p < 0.001; *r* = Spearman Korelasyon Katsayısı; MMSE, Mini Mental State Examinitation; TKS, Tampa Kinesiophobia Scale Score; FRSAS, Fall Risk Self-Assessment Scale.

## 4 Discussion

The results of this study, which planned to assess balance, fall risk, and kinesiophobia in individuals with AD and controls, showed that individuals with AD exhibited worse balance and a higher risk of falls than the control group. AD primarily affects memory and cognitive functions but also impacts motor coordination and spatial awareness. As a result, individuals with AD may struggle to maintain balance and coordination, increasing the likelihood of falls. However, kinesiophobia was found to be similar in AD patients and controls.

Older adults use cognitive control to regulate sensorimotor functioning during balance tasks (Pieruccini-Faria et al., 2019). Therefore, a failure in cognitive functioning can lead to balance impairments and falls in older adults (Mirelman et al., 2012). There is a reciprocal relationship between balance and cognition in older adults, and there is increasing evidence that both are associated with falls (Schäfer et al., 2006). Cognitive control is essential for maintaining balance during dynamic tasks in older adults. Cognitive functions such as attention, memory, and executive control help process sensory information and adapt motor responses accordingly. When these cognitive functions decline, as seen in neurodegenerative diseases like Alzheimer’s or in normal aging, it becomes more challenging to regulate balance (Li et al., 2018).

The Hallmark of AD is progressive cognitive dysfunction, but the concomitant loss of balance, risk of falls and kinesiophobia related to individuals’ independent and safe mobility has been known for many years (Gras et al., 2015). Kluger et al. reported that individuals with mild cognitive impairment (MCI) and AD performed worse on all motor tasks than age-matched asymptomatic controls (Kluger et al., 1997).

Yoon et al. stated that impaired balance is a sign that predicts cognitive decline (Yoon et al., 2020). Studies have shown that the prevalence of gait and balance changes ranges from 9 to 52%, depending on the assessment tool (Puente-González et al., 2020). A meta-analysis found that older adults who successfully maintained a semi-tandem stance for up to 10 s were 28% less likely to experience mild to mild–moderate cognitive impairment within 8 years (Blackwood et al., 2023). Caliskan et al. showed that Alzheimer’s patients show a high risk of falling and balance disorders, measured with the TUG and BERG scales (Caliskan et al., 2023). Köroğlu, stated that according to the Tinetti balance and walking test results, balance and walking problems occur in AD patients (Köroğlu, 2014). Kato-Narita et al. stated that a control group had better balance and less frequent falls than AD patients (Kato-Narita et al., 2011).

The relationship between balance and cognition is bidirectional. Cognitive decline leads to balance issues, and poor balance and falls can further exacerbate cognitive decline. For example, falls can cause injury and fear of falling, which leads to reduced physical activity, further contributing to mental and physical deterioration (Chen et al., 2023).

In the present study, it was found that the mean values of Tinetti balance, Tinetti gait and Tinetti total measurements were lower in individuals with AD compared to a control group. As a result of the study, it was observed that the mean of the Timed Up and Go Test was higher in the AD group than in the Control Group and the duration was longer. According to the Single Leg Standing Test, it was determined that individuals in the AD group could stand on one leg for a shorter time than the control group. These results showed that both static and dynamic balances of individuals with AD were weaker than in the control group. Cognitive impairments in AD affect the ability to process sensory input and regulate motor output. Maintaining balance, both static (standing still) and dynamic (moving), requires the integration of multiple sensory systems (e.g., proprioception, vision, and vestibular input) and motor control. Cognitive decline in AD impairs the brain’s ability to process and respond to this sensory information, leading to deficits in balance. Cognitive decline leads to balance issues, and poor balance and falls can further exacerbate cognitive decline. By preventing falls through early interventions that stem from comprehensive balance assessments, we can reduce hospitalizations and improve the long-term care management of AD patients, making the initial resource investment in assessments cost-effective in the long term. This situation shows that there are impairments in the balance of individuals with mild and moderate AD and that balance and coordination exercises should be included in the treatment of individuals with AD from the early stages.

Atrophy in the cortical motor areas of the brain and the corpus callosum leads to deterioration in motor skills, leading to balance and walking problems, lack of coordination, and slowed movement. As a result, the risk of falling increases (Polat and Kumral, 2010). Segev-Jacubovski et al. (2011) reported in their study that cognitive disorders increase the risk of falling. Borges et al.'s (2015) study showed that individuals with AD have a higher incidence of falling.

The frequency of falls increases with age, and women are at a higher risk compared to men (Lee et al., 2022). Cognitive impairment emerges as an independent risk factor for falls, and consequently, cognitive decline and falls significantly reduce the quality of life in older adults (Baydan et al., 2019). An 8-year prospective cohort study investigating whether changes in cognitive performance can predict falls reported that cognitive performance was associated with falls in adults over 65 years of age; therefore, cognitive performance should be assessed in clinical practice when assessing fall risk (Anstey et al., 2006). Cognitive impairment and fear of falling are risk factors for falls in older people (Borges et al., 2015). A long follow-up study on AD and normal aging reported that falls occurred in 36% of subjects with AD compared to 11% of controls of the same age (Sheridan and Hausdorff, 2007). According to one study, 50% of a group of 157 individuals with AD, 117 of whom were followed for a period of 3 years, either fell or became unable to walk (Buchner and Larson, 1987).

The results of the present study showed that individuals with AD had a higher risk of falling than the control group. In addition, a moderate relationship was found between fall risk, cognitive status, and balance parameters. This result showed that individuals with AD had a higher risk of falls compared to individuals with the same physical characteristics who did not have AD. Many individuals with AD develop a fear of falling due to past falls or the awareness of their increased vulnerability. This fear may cause them to restrict their physical activity, leading to muscle weakness, reduced coordination, and further impairment in balance. This creates a vicious cycle where fear of falling actually increases the risk of future falls. Therefore, environmental and individual protective measures should be addressed early, considering the fall risks of individuals with AD. We also think that caregivers of individuals with AD should be trained. Caregivers of individuals with Alzheimer’s disease (AD) must be trained in a wide range of skills and knowledge to provide adequate care while maintaining their well-being. Education should include information about the disease process, assisting individuals with daily activities such as bathing, dressing, feeding, and toileting, as well as home safety modifications to reduce the risks and hazards of falls.

As AD progresses, patients often experience physical decline, including muscle weakness, reduced coordination, and impaired balance. These physical changes can lead to falls, injuries, or a fear of falling. Once a fall occurs, individuals with AD may develop a heightened fear of movement (kinesiophobia) due to the association of movement with injury (Naugle et al., 2022). Kinesophobia leads individuals to limit and reduce their physical activities (Dabek et al., 2020). Genç and Bilgili found that kinesiophobia was higher in people with low lower extremity strength and balance problems (Genç and Bilgili, 2023). A study found that kinesiophobia is prevalent among older adults with AD and is associated with reduced physical function and increased fear of falling; this fear can lead to decreased physical activity, which exacerbates both physical and cognitive decline in these individuals. The findings suggest that addressing kinesiophobia is crucial for improving mobility and overall health in patients with AD (Naugle et al., 2022). A systematic review found that individuals with cognitive impairments, including those with AD, are particularly vulnerable to developing kinesiophobia after experiencing a fall, which negatively impacts their quality of life (Uysal et al., 2024). Another study noted that individuals with AD who experienced falls reported higher levels of anxiety and kinesiophobia. This psychological response can create a cycle where fear leads to inactivity, which in turn increases the likelihood of future falls and cognitive decline (Dove et al., 2024).

Research has shown that cognitive decline is associated with increased fear avoidance behaviors, including kinesiophobia. The interplay between cognitive impairment and fear of movement can create a feedback loop where each condition exacerbates the other, leading to further deterioration in both physical and cognitive health (Alpalhão et al., 2022). The results of the current study indicate that individuals with Alzheimer’s disease (AD) and a control group have similar levels of kinesophobia. We believe that the similarities in the physical and socio-cultural conditions of both groups contribute to the similarity in their kinesophobia levels. In addition, as a result of the current study, there was a high negative correlation between MMSE and the Timed Up and Go Test, FRSAS, and TKS, indicating that MMSE scores were lower in the control group with a high risk of falls and kinesiophobia. Studies have been conducted on chronic pain, osteoarthritis, and acute-chronic back and neck pain in older adults (Alpalhão et al., 2022). However, research on kinesophobia in individuals with AD is still quite limited. Further studies in this area are needed. When evaluating individuals with AD, kinesophobia should also be a focus, and information should be provided to individuals and their families about the importance, causes, and treatment methods of kinesophobia. Caregivers and healthcare professionals can educate patients and families about the risks of inactivity and the benefits of staying active. This can reduce fear and build trust in safe physical activities.

### 4.1 Limitations of the study

This study provides valuable insights into the multifaceted relationship between balance, fall risk, kinesiophobia, and Alzheimer’s disease. Future studies could investigate how balance, muscle strength, and cognitive function change over time in individuals with AD and how these changes relate to fall risk. These new studies could develop and evaluate interventions aimed at reducing kinesiophobia to improve physical activity levels and reduce fall risk in individuals with AD.

This study has several strengths, including its focus on a clinically important topic, the use of validated tools tailored for the population and cultural context, and its integration of physical and psychological dimensions in AD research. However, limitations such as the small sample size, lack of adjustments for multiple comparisons, and minimal exploration of kinesiophobia reduce its overall impact. Also, the fact that the tests were not applied to all individuals in a counterbalanced order may be a limitation of this study.

Although the sample size of this study was found to be adequate by the power analysis, future studies should be conducted with larger sample sizes. In this study, elderly controls were reached by snowball sampling method. The snowball sampling method relies on the networks and connections within a community to identify potential study participants. One of the primary limitations of snowball sampling is the potential for bias. Since the sample grows based on participants’ referrals, it is heavily influenced by their social networks and preferences. The very nature of snowball sampling means it lacks randomness, a necessary quality of many traditional sampling methods aimed at ensuring representativeness. Participants are chosen based on their connections within a network rather than being randomly selected. Therefore, the use of the snowball sampling method can be considered as a limitation of the study. In the current study, Alzheimer’s disease (AD) patients were not classified and evaluated according to their stages. In future studies, we recommend increasing the sample size and classifying AD patients, as well as including individuals with advanced stages of AD. Additionally, since pain was not assessed in our study, we suggest that future research should evaluate both kinesophobia and pain together.

Additionally, in our study, statistical analyses were performed by controlling age. Some interesting variance related to the variables in our study may have been partialled out when age was controlled. We believe it may be important to conduct future studies considering the effect of age. Also, most of the individuals with AD in our study had moderate AD. Future studies should investigate the relationship between the variables examined in mild AD.

The MMSE is one of the most frequently used screening tools for dementia across the globe, both in research and in clinical practice (Nieuwenhuis-Mark, 2010). However, many studies have demonstrated that the MMSE is affected by educational level, language of administration, and culture (Goudsmit et al., 2018; Nielsen et al., 2012). Studies have attempted to account for some of these confounds by lowering the cutoff score for some populations (Black et al., 1999; Cassimiro et al., 2017). However, evidence suggests that while modifying the cutoff score in the MMSE may increase specificity, it reduces sensitivity (Ostrosky-Solis, 2007). In their meta-analysis study, Maher and Calia pooled existing data that supports the view that many dementia screening tools are not appropriate for illiterate individuals. This finding emphasizes the need for the development and use of tools that are suitable for all individuals, regardless of their literacy ability, education, or cultural background (Maher and Calia, 2021). We also used the MMSE test in our study, but there were individuals who could not read and write. This can be considered a limitation of our study. Further research is required to validate such tools and determine their suitability across various settings.

## 5 Conclusion

By performing comprehensive balance assessments, healthcare providers can identify subtle impairments early, even before the patient experiences a fall. Early identification allows for tailored rehabilitation interventions, such as balance training, physical therapy, and exercise programs, which can prevent further decline and improve physical and cognitive outcomes. Catching problems early may prevent them from worsening, improving long-term health outcomes and reducing the need for more intensive interventions later. While not all individuals with Alzheimer’s disease and older adults will experience kinesiophobia, it is still beneficial to screen for it as part of comprehensive assessments. A personalized approach to care—where psychological and physical interventions are tailored to the patient’s needs—ensures that resources are used efficiently. Not every individual with AD will require interventions for kinesiophobia, but recognizing those who do and providing appropriate support can significantly improve both quality of life and rehabilitation outcomes.

## Data Availability

The original contributions presented in the study are included in the article/supplementary material, further inquiries can be directed to the corresponding author/s.

## References

[ref1] Ağırcan D. (2009). Tinetti Balance and Gait Assessment'ın (Tinetti Denge ve Yürüme Değerlendirmesi) Türkçeye uyarlanması, geçerlilik ve güvenilirliği. Pamukkale Üniversitesi Sağlık Bilimleri Enstitüsü,

[ref2] Allan L. M. Ballard C. G. Rowan E. N. Kenny R. A. (2009). Incidence and prediction of falls in dementia: a prospective study in older people. PLoS One 4:e5521. doi: 10.1371/journal.pone.0005521, PMID: 19436724 PMC2677107

[ref3] Alpalhão V. Cordeiro N. Pezarat-Correia P. (2022). Kinesiophobia and fear avoidance in older adults: a scoping review on the state of research activity. J. Aging Phys. Act. 30, 1075–1084. doi: 10.1123/japa.2021-0409, PMID: 35303715

[ref4] Alzheimer's Association. (2019). Alzheimer’s disease facts and figures. 15(3), 321–387.

[ref5] Anstey K. J. Von Sanden C. Luszcz M. A. (2006). An 8-year prospective study of the relationship between cognitive performance and falling in very old adults. J. Am. Geriatr. Soc. 54, 1169–1176. doi: 10.1111/j.1532-5415.2006.00813.x, PMID: 16913981

[ref6] Arnold C. M. Faulkner R. A. (2007). The history of falls and the association of the timed up and go test to falls and near-falls in older adults with hip osteoarthritis. BMC Geriatr. 7, 1–9. doi: 10.1186/1471-2318-7-17, PMID: 17610735 PMC1936991

[ref7] Baydan M. Caliskan H. Balam-Yavuz B. Aksoy S. Böke B. (2019). Balance and motor functioning in subjects with different stages of cognitive disorders. Arch. Gerontol. Geriatr. 130:110785. doi: 10.1016/j.exger.2019.110785

[ref8] Bertera E. M. Bertera R. L. (2008). Fear of falling and activity avoidance in a national sample of older adults in the United States. Health Soc. Work 33, 54–62. doi: 10.1093/hsw/33.1.54, PMID: 18326450

[ref9] Black S. A. Espino D. V. Mahurin R. Lichtenstein M. J. Hazuda H. P. Fabrizio D.. (1999). The influence of noncognitive factors on themini-mental state examination in olderMexican-Americans. J. Clin. Epidemiol. 52, 1095–1102. doi: 10.1016/S0895-4356(99)00100-6, PMID: 10527004

[ref10] Black A. Wood J. (2005). Vision and falls. Clin. Exp. Optom. 88, 212–222. doi: 10.1111/j.1444-0938.2005.tb06699.x, PMID: 16083415

[ref11] Blackwood J. Amini R. Conti G. Counseller Q. Taylor R. Fayyad D. (2023). Balance performance and grip strength as predictors of cognitive function among community-dwelling older adults in the USA. J. Frailty Sarcopenia Falls 8, 23–31. doi: 10.22540/JFSF-08-023, PMID: 36873827 PMC9975970

[ref12] Blankevoort C. G. Van Heuvelen M. J. Scherder E. (2013). Reliability of six physical performance tests in older people with dementia. Phys. Ther. 93, 69–78. doi: 10.2522/ptj.20110164, PMID: 22976448

[ref13] Borges S. D. M. Radanovic M. Forlenza O. V. (2015). Fear of falling and falls in older adults with mild cognitive impairment and Alzheimer’s disease. Neuropsychol. Dev. Cogn. B Aging Neuropsychol. Cogn. 22, 312–321. doi: 10.1080/13825585.2014.933770, PMID: 24992289

[ref14] Buchner D. M. Larson E. B. (1987). Falls and fractures in patients with Alzheimer-type dementia. JAMA 257, 1492–1495. doi: 10.1001/jama.1987.03390110068028 3820464

[ref15] Caliskan H. Sahin U. K. Baydan M. Ozsurekci C. Aycicek S. Dogrul T.. (2023). Balance performance measured by posturography in mild-moderate Alzheimer's disease: an undervalued assessment. Geriatr. Nurs. 53, 33–39. doi: 10.1016/j.gerinurse.2023.06.019, PMID: 37422938

[ref16] Cassimiro L. Fuentes D. Nitrini R. Yassuda M. S. (2017). Decision-making in cognitively unimpaired illiterate andLow-educated older women: results on the Iowa GamblingTask. Arch. Clin. Neuropsychol. 32, 71–80. doi: 10.1093/arclin/acw080, PMID: 27680089

[ref17] Chen H. Jiang Z. Hu J. Yang X. Gui S. Wang J.. (2023). A bidirectional relationship between cognitive reserve and cognition among older adults in a rural Chinese community: a cross-lagged design. Front. Psychol. 14:1297699. doi: 10.3389/fpsyg.2023.1297699, PMID: 38192390 PMC10773703

[ref18] Crombez G. Vlaeyen J. W. Heuts P. H. Lysens R. (1999). Pain-related fear is more disabling than pain itself: evidence on the role of pain-related fear in chronic back pain disability. Pain 80, 329–339. doi: 10.1016/S0304-3959(98)00229-2, PMID: 10204746

[ref19] Çuhadar D. Sertbaş G. Tutkun H. J. A. (2006). Relationship between level of cognitive functions and activities of daily life at elderly people who live in rest home. Alpha Psychiatry 7, 232–239.

[ref20] Dabek J. Knapik A. Gallert-Kopyto W. Brzek A. Piotrkowicz J. Gasior Z. (2020). Fear of movement (Kinesiophobia)–an underestimated problem in Polish patients at various stages of coronary artery disease. Ann. Agric. Environ. Med. 27, 56–60. doi: 10.26444/aaem/106143, PMID: 32208580

[ref21] Devecchi V. Alalawi A. Liew B. Falla D. (2022). A network analysis reveals the interaction between fear and physical features in people with neck pain. Sci. Rep. 12:11304. doi: 10.1038/s41598-022-14696-8, PMID: 35787648 PMC9253153

[ref22] Dove E. Hewston P. Wang R. H. Patterson K. K. Astel A. J. (2024). Concerns about falling in people with mild cognitive impairment and dementia: a scoping review of exercise interventions. Front. Dement. 3:1456125. doi: 10.3389/frdem.2024.1456125, PMID: 39634256 PMC11615571

[ref23] Dyer A. H. Lawlor B. Kennelly S. P. (2020). Gait speed, cognition and falls in people living with mild-to-moderate Alzheimer disease: data from NILVAD. BMC Geriatr. 20, 117–110. doi: 10.1186/s12877-020-01531-w, PMID: 32228468 PMC7106668

[ref24] Fried L. P. Tangen C. M. Walston J. Newman A. B. Hirsch C. Gottdiener J.. (2001). Frailty in older adults: evidence for a phenotype. J. Gerontol. 56, 146–156. doi: 10.1093/gerona/56.3.M146 11253156

[ref25] Genç F. Z. Bilgili N. (2023). Evaluation of Kinesiophobia and related factors in elderly people living in a nursing home. Balıkesir J. Health Sci. 12, 294–303. doi: 10.53424/balikesirsbd.1140101

[ref26] Goubran M. Farajzadeh A. Lahart M. Bilodeau M. Boisgontier M. P. (2024). Kinesiophobia and physical activity: a systematic review and meta-analysis. medRxiv. doi: 10.1101/2023.08.17.23294240 PMC12207065 40188486

[ref27] Goudsmit M. van Campen J. Schilt T. Hinnen C. Franzen S. Schmand B. (2018). One size does not fit all: comparative diagnostic accuracy of the Rowland universal dementia assessment scale and the mini mental state examination in a memory clinic population with very low education. Dement. Geriat. Cogn. Disord. Extra 8, 290–305. doi: 10.1159/000490174, PMID: 30323830 PMC6180264

[ref28] Gras L. Z. Kanaan S. F. McDowd J. M. Colgrove Y. M. Burns J. Pohl P. S. (2015). Balance and gait of adults with very mild Alzheimer’s disease. Eur. J. Phys. Rehabil. Med. 38:1. doi: 10.1519/JPT.0000000000000020 PMC4632639 24755691

[ref29] Güngen C. Ertan T. Eker E. Yaşar R. Engin F. (2002). Reliability and validity of the standardized Mini mental state examination in the diagnosis of mild dementia in Turkish population. Turk. Psikiyatri. Derg. 13, 273–281, PMID: 12794644

[ref30] Gürer A. Çırpan F. K. Özlen N. A. (2019). Yaşlı bakım hizmetleri. J. Health Serv. Educ. 3, 1–6. doi: 10.35333/JOHSE.2019.44

[ref9001] Güzel R. İrdesel J. Kutsal Y. G. (2021). Kinesiophobia in older ages. Journal of Continuing Medical Education, 30, 116–125.

[ref31] Hawk C. Hyland J. K. Rupert R. Colonvega M. Hall S. (2006). Assessment of balance and risk for falls in a sample of community-dwelling adults aged 65 and older. Chiropr. Osteopat. 14, 1–8. doi: 10.1186/1746-1340-14-3, PMID: 16441893 PMC1413542

[ref32] Işık A. Tanrıdağ O. (2009). Geriatri Pratiğinde Demans Sendromu. 4(1).

[ref33] Jia L. Du Y. Chu L. Zhang Z. Li F. Lyu D.. (2020). Prevalence, risk factors, and management of dementia and mild cognitive impairment in adults aged 60 years or older in China: a cross-sectional study. Lancet Public Health 5, e661–e671. doi: 10.1016/S2468-2667(20)30185-7, PMID: 33271079

[ref34] Kato-Narita E. M. Nitrini R. Radanovic M. (2011). Assessment of balance in mild and moderate stages of Alzheimer's disease: implications on falls and functional capacity. Arq. Neuropsiquiatr. 69, 202–207. doi: 10.1590/S0004-282X2011000200012, PMID: 21537561

[ref35] Kaya T. Göksel Karatepe A. Avcı A. Günaydın R. (2012). Yaşlılarda düşme korkusu ve düşmeye karşı yetkinlik. Türk. Geriatri. Dergisi 15:265.

[ref36] Kluger A. Gianutsos J. G. Golomb J. Ferris S. H. George A. E. Franssen E.. (1997). Patterns of motor impairment in normal aging, mild cognitive decline, and early Alzheimer'Disease. J. Gerontol. B Psychol. Sci. Soc. Sci. 52, P28–P39. doi: 10.1093/geronb/52B.1.P28 9008673

[ref37] Kori S. (1990). Kinesiophobia: a new view of chronic pain behavior. Clin. Orthop. Relat. Res. 3, 35–43.

[ref38] Köroğlu Ç. (2014). Gait and balance assessment in Alzheimer's patients: a comparative study (Master's thesis, Pamukkale University health Sciences institute). Denizli, Turkey.

[ref39] Lee B.-K. So W.-Y. Kang H.-J. (2022). Analysis of fall events, physical fitness, and gait speed according to fall risk in older Korean women. Healthcare (Basel) 10:1936. doi: 10.3390/healthcare10101936, PMID: 36292383 PMC9601472

[ref40] Lethem J. Slade P. Troup J. Bentley G. (1983). Outline of a fear-avoidance model of exaggerated pain perception—I. Behav. Res. Ther. 21, 401–408. doi: 10.1016/0005-7967(83)90009-8, PMID: 6626110

[ref41] Li K. Z. H. Bherer L. Mirelman A. Maidan I. Hausdorff J. M. (2018). Cognitive involvement in balance, gait and dual-tasking in aging: a focused review from a neuroscience of aging perspective. Front. Neurol. 9:913. doi: 10.3389/fneur.2018.00913, PMID: 30425679 PMC6219267

[ref42] Liu Y. Ye F. Liu J. Yu J. Fan Y. Yang Q. (2024). Study of factors influencing early Kinesiophobia in older patients after coronary artery bypass grafting in China. Heart Surgery Forum 27, E504–E510. doi: 10.59958/hsf.7377

[ref43] Maher C. Calia C. (2021). The effect of illiteracy on performance in screening tools for dementia: a meta-analysis. J. Clin. Exp. Neuropsychol. 43, 945–966. doi: 10.1080/13803395.2022.2040433, PMID: 35200100

[ref9002] Miller R. R. Kori S. H. Todd D. D. (1991). The Tampa Scale: a measure of kinisophobia. The Clinical journal of pain, 7:51.

[ref9004] Mirelman A. Herman T. Brozgol M. Dorfman N. Sprecher E. Schweiger A.. (2012). Executive function and falls in older adults: new findings from a five-year prospective study link fall risk to cognition. PLoS One 7:e40297. doi: 10.1371/journal.pone.0040297, PMID: 22768271 PMC3386974

[ref46] Naugle K. Blythe C. Naugle K. E. Keith N. Riley Z. A. (2022). Kinesiophobia predicts physical function and physical activity levels in chronic pain-free older adults. Front. Pain Res. 3:874205. doi: 10.3389/fpain.2022.874205, PMID: 35571145 PMC9091550

[ref47] Nielsen T. R. Vogel A. Gade A. Waldemar G. (2012). Cognitive testing in non-demented Turkishimmigrants–comparison of the RUDAS and the MMSE. Scand. J. Psychol. 53, 455–460. doi: 10.1111/sjop.12018, PMID: 23170863

[ref48] Nieuwenhuis-Mark R. E. (2010). The death knollfor the MMSE: has it outlived its purpose? J. Geriat. Psychiatry Neurol. 23, 151–157. doi: 10.1177/0891988710363714, PMID: 20231732

[ref49] Ostrosky-Solis F. (2007). “Educational effects on cognitive func-tions: brain reserve, compensation, or testing bias?” in International handbook of cross-cultural neuropsychology. eds. Uzzell B. P. Pontón M. O. Ardila A. (Laurence Erlbaum Associates), Mahwah, New Jersey. 215–225.

[ref50] Özsoy O. (2014). İktisatçılar ve İstatistikçiler İçin İstatistik. Ankara: Pegem Akademi.

[ref51] Passeri E. Elkhoury K. Morsink M. Broersen K. Linder M. Tamayol A.. (2022). Alzheimer’s disease: treatment strategies and their limitations. Int. J. Mol. Sci. 23:13954. doi: 10.3390/ijms232213954, PMID: 36430432 PMC9697769

[ref52] Pieruccini-Faria F. Lord S. R. Toson B. Kemmler W. Schoene D. (2019). Mental flexibility influences the association between poor balance and falls in older people - a secondary analysis. Front. Aging Neurosci. 11:133. doi: 10.3389/fnagi.2019.00133, PMID: 31263408 PMC6584815

[ref53] Podsiadlo D. Richardson S. J. (1991). The timed “up & go”: a test of basic functional mobility for frail elderly persons. J. Am. Geriatr. Soc. 39, 142–148. doi: 10.1111/j.1532-5415.1991.tb01616.x, PMID: 1991946

[ref54] Polat F. Kumral E. (2010). Normal and pathologic brain aging. Ege J. Med. 49, 3–10.

[ref55] Puente-González A. S. Sanchez-Gonzalez F. Hernández-Xumet J. E. Sánchez-Sánchez M. C. Barbero-Iglesias F. J. Mendez-Sanchez R. (2020). Short and medium-term effects of a multicomponent physical exercise program with a Mediterranean diet on bone mineral density, gait, balance, and fall risk for patients with Alzheimer disease: randomized controlled clinical trial study protocol. Medicine (Baltimore) 99:e22385. doi: 10.1097/MD.0000000000022385, PMID: 32957420 PMC7505369

[ref56] Ries J. D. Hutson J. Maralit L. A. Brown M. B. (2015). Group balance training specifically designed for individuals with Alzheimer disease: impact on berg balance scale, timed up and go, gait speed, and mini-mental status examination. J. Geriatr. Phys. Ther. 38, 183–193. doi: 10.1519/JPT.0000000000000030, PMID: 25621384

[ref57] Rostagno A. A. (2022). Pathogenesis of Alzheimer’s disease. Int. J. Mol. Sci. 24:107. doi: 10.3390/ijms24010107, PMID: 36613544 PMC9820480

[ref9003] Savaş S. Akçiçek F. (2010). Comprehensive geriatric assessment. Ege Journal of Medicine, 49, 19–30

[ref58] Schäfer S. Huxhold O. Lindenberger U. (2006). Healthy mind in healthy body? A review of sensorimotor–cognitive interdependencies in old age. Eur. Rev. Aging Phys. Act. 3, 45–54. doi: 10.1007/s11556-006-0007-5

[ref59] Scheffer A. C. Schuurmans M. J. van Dijk N. van der Hooft T. de Rooij S. E. (2008). Fear of falling: measurement strategy, prevalence, risk factors and consequences among older persons. Age Ageing 37, 19–24. doi: 10.1093/ageing/afm169, PMID: 18194967

[ref60] Segev-Jacubovski O. Herman T. Yogev-Seligmann G. Mirelman A. Giladi N. Hausdorff J. M. (2011). The interplay between gait, falls and cognition: can cognitive therapy reduce fall risk? Expert. Rev. Neurother. 11, 1057–1075. doi: 10.1586/ern.11.69, PMID: 21721921 PMC3163836

[ref61] Sertel M. Şimşek T. Yümin E. Aras B. (2020). Determination of the validity and reliability of the Turkish version of the self-rated fall risk questionnaire in older individuals. Physiotherapy Quart. 28, 50–55. doi: 10.5114/pq.2020.95775, PMID: 39649281

[ref62] Sheridan P. L. Hausdorff J. M. (2007). The role of higher-level cognitive function in gait: executive dysfunction contributes to fall risk in Alzheimer’s disease. Dement. Geriatr. Cogn. Disord. 24, 125–137. doi: 10.1159/000105126, PMID: 17622760 PMC3163262

[ref63] Stevenson T. J. Garland S. (1996). Standing balance during internally produced perturbations in subjects with hemiplegia: validation of the balance scale. Arch. Phys. Med. Rehabil. 77, 656–662. doi: 10.1016/S0003-9993(96)90004-0, PMID: 8669991

[ref65] Thrane G. Joakimsen R. M. Thornquist E. (2007). The association between timed up and go test and history of falls: the Tromsø study. BMC Geriatr. 7, 1–7. doi: 10.1186/1471-2318-7-1, PMID: 17222340 PMC1781456

[ref66] Tinetti M. E. (1986). Performance-oriented assessment of mobility problems in elderly patients. J. Am. Geriatr. Soc. 34, 119–126. doi: 10.1111/j.1532-5415.1986.tb05480.x, PMID: 3944402

[ref67] Tuncay S. U. Özdinçler A. R. Erdincler D. S. (2011). The effect of risk factors for falls on activities of daily living and quality of life in geriatric patients. J. Nutr. Health Aging 14, 245–252.

[ref68] Uysal I. Özden F. Özkeskin M. Benzer Z. Işık E. I. (2024). Exercise barriers in older individuals with Alzheimer’s disease: a cross-sectional study. Medicina (Kaunas) 60:1510. doi: 10.3390/medicina60091510, PMID: 39336551 PMC11434187

[ref69] Vlaeyen J. W. Crombez G. Linton S. (2016). The fear-avoidance model of pain. Pain 157, 1588–1589. doi: 10.1097/j.pain.0000000000000574, PMID: 27428892

[ref70] Vlaeyen J. W. Kole-Snijders A. M. Boeren R. G. Van Eek H. (1995). Fear of movement/(re)injury in chronic low back pain and its relation to behavioral performance. Pain 62, 363–372. doi: 10.1016/0304-3959(94)00279-N, PMID: 8657437

[ref71] Vranceanu A. M. Choukas N. R. Rochon C. A. Duarte B. Pietrzykowski M. O. McDermott K.. (2023). Addressing the chronic pain-early cognitive decline comorbidity among older adults: protocol for the active brains remote efficacy trial. JMIR Res. Protoc. 12:e47319. doi: 10.2196/47319, PMID: 37768713 PMC10570897

[ref73] Yilmaz Ö. T. Yakut Y. Uygur F. Uluğ N. (2011). Turkish version of the Tampa Scale for Kinesiophobia and its test-retest reliability. Fizyoter Rehabil, 22, 44–49.

[ref74] Yoon B. Choi S. H. Jeong J. H. Park K. W. Kim E.-J. Hwang J.. (2020). Balance and mobility performance along the Alzheimer’s disease spectrum. J. Alzheimers Dis. 73, 633–644. doi: 10.3233/JAD-190601, PMID: 31815691

